# Importance of medication reconciliation in cancer patients

**DOI:** 10.1186/s40545-021-00379-8

**Published:** 2021-11-29

**Authors:** Ali Elbeddini, Anthony To, Yasamin Tayefehchamani, Cindy Xin Wen

**Affiliations:** 1 Chairman of the Pharmacy Department, Winchester District Memorial Hospital, 566 Louise Street, Winchester, ON KK0C2K0 Canada; 2 Leslie Dan Faculty of Pharmacy, University of Toronto, 144 college st, Toronto, M5S 3M2 Canada

**Keywords:** Medication reconciliation, Oncology, Cancer

## Abstract

Cancer patients are a complex and vulnerable population whose medication history is often extensive. Medication reconciliations in this population are especially essential, since medication discrepancies can lead to dire outcomes. This commentary aims to describe the significance of conducting medication reconciliations in this often-forgotten patient population. We discuss additional clinical interventions that can arise during this process as well. Medication reconciliations provide the opportunity to identify and prevent drug–drug and herb–drug interactions. They also provide an opportunity to appropriately adjust chemotherapy dosing according to renal and hepatic function. Finally, reconciling medications can also provide an opportunity to identify and deprescribe inappropriate medications. While clinical impact appears evident in this landscape, evidence of economic impact is lacking. As more cancer patients are prescribed a combination of oral chemotherapies, intravenous chemotherapies and non-anticancer medications, future studies should evaluate the advantages of conducting medication reconciliations in these patient populations across multiple care settings.

## Introduction

Cancer patients have many risk factors that characterize them as a vulnerable population. A large portion of cancer patients are elderly, a population with higher incidence rates of multiple comorbidities. Polypharmacy, frequently defined as the use of 5 or more medications, is also more prevalent in cancer patients. With polypharmacy, there is also an increased risk of drug interactions as well as adverse drug reactions. In addition to existing medications, cancer patients are frequently administered chemotherapy, hormone therapy, biological agents and supportive therapies, thereby causing polypharmacy in this population. Moreover, cancer is heavily taxing on the body and can cause organ failure and systemic deterioration, increasing the risk of complications throughout its progression. Due to the complex nature of cancer patients, it is essential to ensure their medications are optimized to achieve the best patient care outcome.

A top priority across all health care settings is providing patient-centered care. When making patient care decisions, it is important to gather a complete medical history that can be shared across all health care providers involved to ensure a seamless transition across different settings. In complex patients who have multiple comorbidities and are taking several medications, it is especially crucial to identify discrepancies and to ensure medication safety. Medication reconciliation is a process that specifically seeks to improve medication safety across different points of care. It consists of obtaining a comprehensive list of all medications taken by a patient and comparing it to the current drug regimen to identify and resolve any discrepancies. Although many healthcare professionals can offer assistance in the process, pharmacists are often considered the best suited role to perform the medication reconciliation. Current literature surrounding the benefit of medication reconciliations is disproportionate as there are many studies done in other populations, while only a few done in oncology patients. The aim of this commentary is to provide an overview of the importance of conducting medication reconciliations in an often-forgotten patient population, cancer patients.

### Significance of conducting medication reconciliation in cancer patients

Medication reconciliation is an essential process that provides value in clinical practice. Its clinical impact on cancer patients was recently evaluated in a systematic review by Herledan et al. [1]. They evaluated 14 studies and found that medication reconciliation practices identified discrepancies and other drug-related problems in up to 88% and 94% of patients, respectively [1]. The most frequent discrepancies and medication errors identified were drug omission, drug additions, and dosage errors [1]. A few studies also reported that discrepancies were found to be more frequent in cancer patients. Kraus et al. found that of 63.6% of cancer patients (*n* = 33) presented with at least one discrepancy compared to 52.5% in the overall study population (*n* = 200) [2]. Another study found that the incidence of at least one discrepancy at admission was 80% in hospitalized patients admitted for cancer-related causes, compared to 53.6% for surgical causes, 74.1% for organ dysfunction, and 57.3% for other causes [3]. These studies highlight the increased risk of medication discrepancies in cancer patients and the need to focus on this population to ensure medication safety.

Medication reconciliations are vital at all interfaces of care from admission to discharge and in both hospital and ambulatory cancer patients. Clinical benefits have been described in multiple settings. In a study with short-term hospitalized cancer patients, 64 interventions were performed after medication reconciliations were conducted in 95 patients [4]. In an ambulatory oncology setting, Weingart et al. detected discrepancies in medication lists, such as medication errors and omissions, in as many as 81% of patients [5]. One study compared conducting medication reconciliations in each of three chemotherapy cycles to only conducting a single reconciliation in the third cycle. They found that conducting medication reconciliations at each cycle resulted in a 26% reduction in reconciliation errors that reached the patient (4% vs 30%) [6]. Conducting medication reconciliations were also shown to have positive outcomes on patient discharge readiness from hospital. In the study by Duffy et al. patient readiness for discharge into a home hospice was higher when a care initiative involving medication reconciliations was conducted [7]. Regarding long-term effects of medication reconciliations, studies in the cancer population is limited. One study by Nguyen et al. found inconclusive results, where only a subgroup of cancer patients had a reduction in hospital readmissions [8]. However, in a meta-analysis by Mekonnen et al. medication reconciliations conducted in adult hospitalized patients from various units were associated with a significant reduction in hospital readmissions and emergency department visits [9]. Further evaluations are necessary to determine if this association can be validated specifically in the cancer population.

The economic impact of conducting medication reconciliations in cancer patients is currently lacking. One study, reviewing gynaecological oncology patients, does show promising results although it was not a comparative evaluation. Son et al. conducted a cost–benefit analysis surrounding the use of medication reconciliations at admission [4]. Benefits were described as cost savings estimated from unused drugs from deprescribing and avoided hospitalizations due to prevented adverse drug events, while costs were associated with pharmacist labour cost [4]. They calculated a benefit:cost ratio of 2.31:1 associated with conducting medication reconciliations [4]. More economic evaluations, ideally comparative studies, should be conducted to support the standardized use of medication reconciliations in cancer patients.

A limitation in the medication reconciliation process is the inconsistencies surrounding its methodology in practice. The systemic review by Herledan et al. found that some practices only conducted medication reconciliations at discharge but not at admission, some did not specify sources of information used to complete medication lists, and some did not interview patients at all to obtain medication histories [1]. To establish uniformity across practices, health care providers should refer to the Standard Operating Protocol for Medication Reconciliations that the World Health Organization (WHO) developed [10]. This protocol is created in accordance to the High 5s Project, which aims to standardize activities to ensure patient safety around the world [10].

**Fig. 1:**
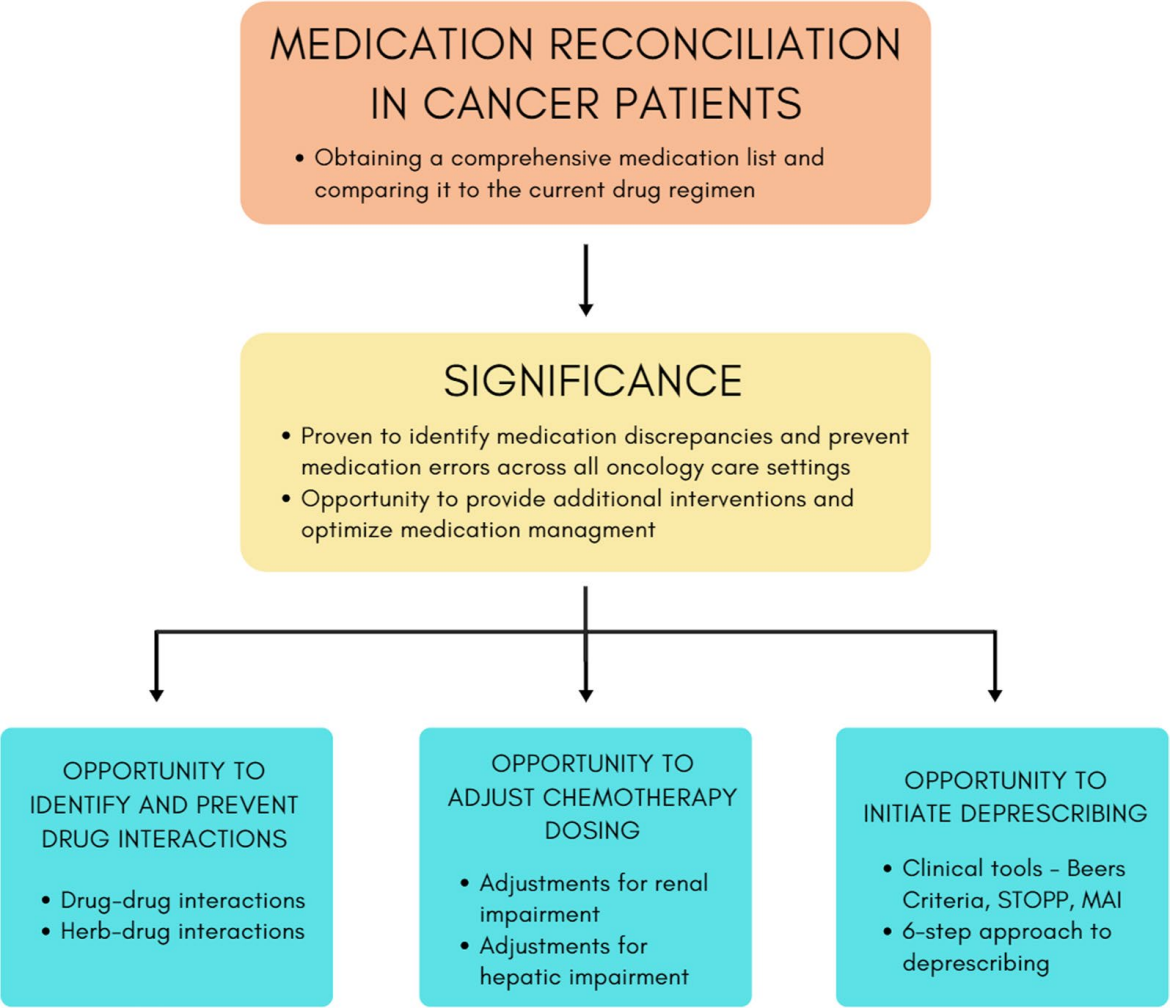
Importance of medication reconciliations in cancer patients

Nevertheless, medication reconciliations have been consistently shown to effectively reduce medication errors in cancer patients in a variety of care settings. The current literature also emphasizes that medication reconciliations enable pharmacists to perform additional interventions, such as providing education on adverse drug reactions and appropriate use of medications [1]. This commentary describes other opportunities that can also be provided by conducting medication reconciliations to optimize medication therapy in cancer patients, namely, identifying and preventing drug interactions, adjusting chemotherapy dosing as well as initiating deprescribing (Fig. 1).

### Opportunity to identify and prevent drug interactions

Polypharmacy has been reported to be prevalent in 11–96% of elderly oncology patients [11]. Cancer patients may be exposed to chemotherapy, hormonal therapy, and supportive agents in addition to their medications used to treat existing medical conditions. The use of multiple medications is associated with an increased risk of drug–drug interactions. Now, with over 25% of all cancer treatments administered orally, it is imperative to also maintain optimal medication safety in the community setting [6]. Medication reconciliations provide an opportunity to detect drug interactions with cancer treatments and to make appropriate clinical interventions to ensure medication safety. We will explore some common drug–drug interactions with chemotherapies and hormonal therapies in cancer patients that may be detected during medication reconciliations.

### Drug–drug interactions with anticancer agents

One particularly controversial and important interaction involves tamoxifen and selective serotonin reuptake inhibitor (SSRI) antidepressants. Tamoxifen is an estrogen antagonist that is used for the treatment of breast cancer in women with estrogen receptor positive tumors. Tamoxifen is generally used for 5–10 years, where it has shown to decrease disease recurrence as well as death due to breast cancer [12, 13]. Tamoxifen is converted to its active metabolite, endoxifen, by the highly polymorphic cytochrome P450 isoenzyme 2D6 (CYP2D6) to exert its therapeutic effects [14]. Consequently, it is hypothesized that drugs that inhibit CYP2D6, such as SSRIs, may interfere with the bioactivation of tamoxifen and result in reduced clinical benefit and treatment failure. This is concerning, since up to 25% of breast cancer patients report a depressive disorder and 24–40% of tamoxifen users are concurrently prescribed an antidepressant [15, 16]. The current literature evaluating the clinical significance of this interaction appear to have mixed conclusions. One population cohort study found that breast cancer patients taking tamoxifen and paroxetine concomitantly had increased risk of death [17]. Other SSRIs with milder CYP2D6 inhibitor potential did not show this association [17]. Another study involving 14,532 women with breast cancer found no difference in mortality between those taking tamoxifen with a potent CYP2D6 inhibitor and those taking tamoxifen with other SSRIs [18]. However, a limitation in this study is that the follow-up time of ~ 2 years may have been too short to observe any differential survival benefit [18]. Regarding the risk of recurrence, Hague et al. found no increased risk of breast cancer recurrence in breast cancer survivors who received concurrent tamoxifen and antidepressants [19]. While the data may be inconclusive, it is advisable to still prescribe antidepressants with caution in patients receiving tamoxifen. A guiding principle is to selectively avoid antidepressants that are known to inhibit CYP2D6, such as paroxetine, fluoxetine, duloxetine and bupropion.

Tyrosine kinase inhibitors (TKIs) are another class of oral anticancer agents that have rapidly become part of treatment guidelines for several cancers, such as leukemia, renal, lung, pancreatic, etc. They work by interfering with growth factor signalling which leads to tumor cell death. Acid suppressing agents that reduce gastric pH, such as proton pump inhibitors (PPIs) and histamine_2_–receptor antagonists (H2RAs), have been shown to affect the pharmacokinetics of TKIs by reducing absorption, area under the curve (AUC), Cmax, and bioavailability [20]. The fact that approximately 23% of cancer patients are reported to receive TKIs and PPIs concomitantly raises concerns around the clinical significance of this interaction [21]. A few reviews evaluated the clinical effect of this drug interaction and a similar consensus of mixed evidence was found [22–24]. For example, one study showed a negative effect on survival with concomitant use of acid suppressing agents and erlotinib [25]. In another study, no association with survival was found in patients taking acid suppressors with sunitinib [26]. These studies highlight that not all TKIs may be affected by acid suppressing medications and that it is difficult to provide concrete guidelines due to the conflicting literature. Nevertheless, caution should be used when prescribing acid suppressing therapy to cancer patients. The general consensus remains to avoid the combination of acid suppressing agents and TKIs if possible [22]. If there is a valid indication for an acid suppression medication, there are practical recommendations to manage the interaction between these agents and TKIs. Enteric coated PPIs have a delayed onset of action of around 3–4 h. To target this window of acidity, TKIS should be taken at least 2 h before the PPI to minimize any pharmacokinetic interaction [22]. If H2RAs are to be used, TKIs should be taken at least 2 h before or 10 h after H2RA intake [22].

The management of anticoagulants in cancer patients is also complex. Patients with cancer have been shown to have a four to eightfold higher risk of developing venous thromboembolisms (VTEs) than the general population [27, 28]. Their increased risk may be due to specific cancer types, cancer therapies, hypercoagulable state, as well as individual factors, such as advanced age [23, 29]. In addition, there appears to be an association with atrial fibrillation (AF) and cancer. It is estimated that up to 25% of overall AF patients have a comorbid cancer diagnosis [29]. However, a causal relationship between AF and cancer remains unclear. Nevertheless, cancer patients require anticoagulants to manage VTEs and stroke prevention in AF. One important anticoagulant that can have interactions with chemotherapy agents is warfarin. This anticoagulant works by suppressing the synthesis of clotting factors through Vitamin K antagonism. Warfarin is also metabolized by CYP2D9, hence medications that inhibit CYP2D9 are a concern. For example, warfarin has been shown to interact with tamoxifen, capecitabine, abiraterone, erlotinib, ceritinib, etc., whereby the interaction causes increased patient exposure to warfarin, which may lead to a higher international normalized ratio (INR) and increased risk of bleeding [23, 24]. Current general recommendations for anticoagulation in cancer patients is to use low-molecular weight heparins for treatment of VTE, and warfarin for stroke prevention in AF [29]. While warfarin remains a high risk drug, there is emerging evidence for the use of direct oral anticoagulants (DOACs) instead. In the ARISTOTLE trial, apixaban showed superior safety and efficacy compared to warfarin in 157 cancer patients [30]. Similar results were seen in observational cases with rivaroxaban [31]. DOACs have less drug interactions than warfarin but should be avoided with cancer therapies that are strong P-gp inducers or inhibitors [29]. If warfarin is necessary for certain cancer patients, it is important to closely monitor INR and signs of bleeding. When conducting a medication reconciliation, it is crucial to identify potential drug interactions and to optimize anticoagulation strategies specific to each cancer patient.

### Herb–drug interactions with anticancer agents

Complementary and alternative medication (CAM) are often used in cancer patients. A systematic review found the prevalence of using vitamin or dietary supplements was reported to be 64% to 81% in adult cancer patients compared to approximately 50% in the general adult population [32]. Another study found that the prevalence of CAM in senior adult oncology patients was 26.5% [33]. In the pediatric cancer patients, one study reported the prevalence of CAM use to be 6–91% [34]. Since the prevalence is so high in the cancer population, Herb–drug interactions are of great concern, especially since they may interfere with cancer treatment regimens. Theoretically, many herbs may interfere with anticancer agents through pharmacokinetic and pharmacodynamic interactions. For example, antioxidant supplements have the potential to interact with certain chemotherapies [35]. Agents such as anthracyclines, platinum, and alkylating agents work by generating free radicals and antioxidants could potentially counteract their effects [35]. Although clinical significance is still uncertain, patients are advised to avoid herbs and supplements with antioxidant effects during cancer treatment [26]. Other herbal products that have potential to interact with anticancer agents include green tea, evening primrose, turmeric, ginger, and medicinal mushrooms [35, 36]. Some herbal products have been shown to have clinically relevant interactions. One case report discussed the interaction between echinacea, a popular immunomodulatory supplement, and etoposide, where concomitant use decreased a patient’s platelet count significantly compared to taking etoposide alone [37]. Another case report noted an interaction between ginseng and imatinib. A patient who has been taking imatinib for 7 years began to display symptoms of hepatotoxicity after ginseng consumption, which then resolved upon discontinuation of ginseng [38]. In addition, 2 studies found that St. John’s wort, a common herbal supplement used for depression, decreased plasma concentration of imatinib by around 30%, which could potentially risk therapeutic failure [39, 40]. These examples highlight the potential risks that could occur with chemotherapy interactions. In a study by Chun et al. they found that vitamins and minerals accounted for the largest portion of additions and modifications found through pharmacist-led medication reconciliations [41]. Without medication reconciliations, it can be easy to miss herbal products in a patient’s medication list. It is important to identify the use of herbal supplements in cancer patients and to detect possible clinical interactions. Drug-interaction databases, such *Lexi-Interact* and *Natural Medicine*, a natural health product specific database, are validated resources that may be used. As there is still uncertainty regarding the clinical impact of herb–drug interactions, it is advisable to be cautious and avoid the concomitant use of anticancer agents and herbal products until further research validates the safety of concomitant use.

### Opportunity to adjust chemotherapy dosing

Kidney damage such as acute kidney injury (AKI) and chronic kidney disease (CKD) can occur in cancer patients due to cancer complications as well as chemotherapy induced nephrotoxicity. AKI has many causes, such as volume depletion, light chain cast nephropathy, tumor lysis syndrome, tumor infiltration, as well as thrombotic microangiopathy [42]. CKD can also be caused by prior episodes of AKI, chronic obstructive nephropathy, and kidney irradiation [42]. In a population-based study from 2007 to 2014, nearly 1 in 10 cancer patients had an incidence of AKI [43]. In another study looking at CKD, 30% of cancer patients had an eGFR of 45 to 59 mL/min/1.73 m^2^, and 8.3% had an eGFR of < 45 mL/min/1.73 m^2^ [44]. Since the incidence of kidney damage is so high, many patient’s chemotherapies may need to be dose adjusted to reduce the risk of toxicities and adverse reactions. Not only is it important to assess kidney function and dose adjustments in patients receiving intravenous chemotherapies in hospital, but also in outpatients receiving oral chemotherapies in the community. For example, guidelines from Cancer Care Ontario (CCO) suggest that capecitabine, a common oral chemotherapy agent, should be dosed at 75% if creatinine clearance (CrCL) is 30 to 50 ml/min and discontinued if CrCL < 30 mL/min [45]. If doses are not adjusted appropriately for capecitabine, patients may have increased risk of gastrointestinal, dermatological toxicity, neurotoxicity, and hyperbilirubinemia [45]. This highlights the importance of conducting medication reconciliations during each cycle of chemotherapy to ensure doses are ordered appropriately for all cancer patients.

Acute and chronic liver damage can also be present in cancer patients for several reasons. Acute liver failure can be caused by viral infection, drugs and toxins, autoimmune hepatitis, ischemia as well as tumor infiltration [46]. Chronic liver injury, commonly referred to as cirrhosis, is mainly caused by alcoholic liver disease and hepatitis C [47]. Hepatotoxic chemotherapies can further decrease liver function in a dose independent manner. The specific prevalence of hepatic impairment in cancer patients is currently unknown. Nonetheless, it is important to monitor liver function in cancer patients, since liver impairment can alter the pharmacokinetic profile of chemotherapies which can lead to subtherapeutic levels and treatment failure or supratherapeutic levels and drug toxicity. A liver panel, including aminotransferases and bilirubin, should be conducted before each administration of chemotherapy, since some may need dose adjustments for hepatic impairment. For example, CCO suggests a dose reduction of 25% if bilirubin levels are 1–2 × upper limit of normal (ULN) for daunorubicin, a commonly used agent for leukemia [48]. If bilirubin levels are 2–4 × ULN, a 50% dose reduction is suggested and if bilirubin levels are > 4 × ULN, then the dose should be omitted for that cycle [39]. Other agents, such as docetaxel, may require dose adjustments based on other liver parameters, such as AST, ALT, bilirubin, and alkaline phosphate levels [49]. These examples highlight the complexity with dosing chemotherapies.

The examples highlighted here are specific to chemotherapies; however, dose adjustments may be appropriate for all drugs that may be excreted through the kidney or metabolized by the liver. In an oncology perspective, medication reconciliations provide opportunities to assess chemotherapy medications and to ensure they are appropriately dosed, since dosing discrepancies can have major consequences in this population.

### Opportunity to deprescribe potentially inappropriate medications

As stated earlier, polypharmacy, commonly described as the use of five or medications, has been shown to be prevalent in 11–96% of elderly cancer patients [11]. While polypharmacy may have therapeutic benefit, it is also associated with adverse drug reactions, increased drug–drug interactions, prescribing errors, negative health outcomes, frailty, functional decline, and mortality [11, 50]. Taking a high number of medications also increases the risk of being on potentially inappropriate medications (PIMs) [51]. PIMs are described as medications that lack appropriate indications, have risks that outweigh therapeutic benefit, or those that can potentially interact with other medications [11]. The prevalence of PIMs has been shown to be quite high in cancer patients, where it has been reported to be between 41 and 52% [52, 53]. PIMs are problematic for elderly cancer patients, since they are associated with postoperative delirium and readmission and could potentially be associated with lower progression-free survival and higher mortality [51]. Medication reconciliations provide an up-to-date comprehensive medication list, where health care providers can identify PIMs and to potentially deprescribe them appropriately to optimize medication safety in cancer patients.

There are many tools available to help identify PIMs, including the Beers Criteria, Screening Tool for Older People’s Prescriptions (STOPP), and the Medication Appropriateness Index (MAI). The Beers Criteria, recently updated in 2019, provides a list of potentially problematic medications to avoid in elderly patients 65 and older [54]. The STOPP criteria is used to identify PIMs in the elderly, including drugs and doses to avoid that can cause drug–drug interactions, risk of falls and duplicate therapy [55]. Another tool is the MAI, which uses ten questions to facilitate the use of clinical judgement in assessing medication appropriateness [56]. There is evidence that use of these tools can help identify PIMs in cancer patients, leading to clinical interventions. In one study, the overall prevalence of PIMs was 51% in 234 ambulatory senior cancer patients, where 38% were identified by the STOPP criteria and 40% were identified by the 2012 Beers criteria [53]. The most prevalent PIMs found were benzodiazepines, GI medications, nonsteroidal anti-inflammatory drugs, and antiplatelet medications [53]. In another study, the 2015 Beers Criteria, STOPP and MAI were used to identify PIM use in 26 cancer patients aged 65 and over. They identified 119 PIMs in total, where 73% of PIMs were deprescribed, such as vitamins/minerals, antihypertensives, statins, benzodiazepines, NSAIDS, and proton pump inhibitors [57]. Afterwards, two-thirds of those patients reported a reduction in symptoms after deprescribing [57]. This study highlights the effectiveness of deprescribing as an intervention once PIMs have been identified. However, there are limitations to these clinical tools in the cancer population. Some medications identified as inappropriate through the Beers Criteria may be necessary for cancer patients. For example, medications deemed inappropriate such as metoclopramide, haloperidol, anticholinergics and benzodiazepines may have a role in treatment of chemotherapy induced nausea and vomiting [50]. To address this issue, Miller et al. proposed a strategy, where clinical judgement with the MAI can be used after Beer’s Criteria has been applied to assess medications that are questionable [58].

**Fig. 2:**
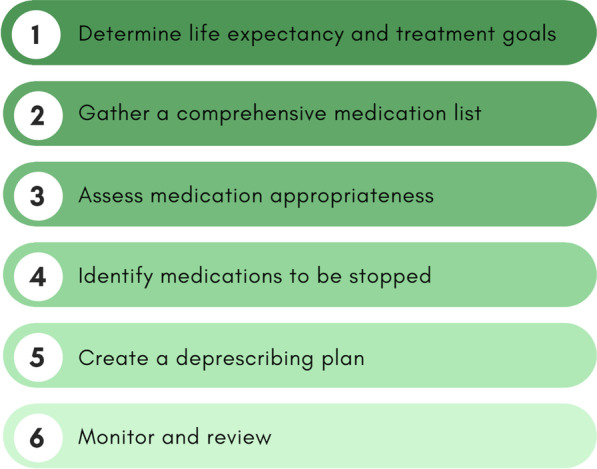
Six-step approach to deprescribing in elderly cancer patients

Deprescribing medications can be a challenge especially in complex populations, such as cancer patients. As a result, this process often requires a multidisciplinary team. A six-step approach to deprescribing in older cancer patients has been developed to assist health care providers with the process (Fig. 2) [59]. Step one involves determining the patient’s life expectancy and treatment goals. Step two involves gathering a comprehensive list of all medications. Step three assesses each medication appropriateness according to individual life expectancy and treatment goals. Step four includes identifying medications to be stopped. Step five involves creating a deprescribing plan. Finally, step six entails monitoring and reviewing events following interventions. Once inappropriate medications have been identified, there are several guidelines, such as those available at www.deprescribing.org, to help create a deprescribing plan. Ultimately, to deprescribe PIMs in cancer patients, a comprehensive list of medications must first be obtained. This key step in the process highlights the importance of conducting medication reconciliation in this patient population, where deprescribing can then be introduced.

### Medication reconciliations across multiple pharmacy settings

**Fig. 3:**
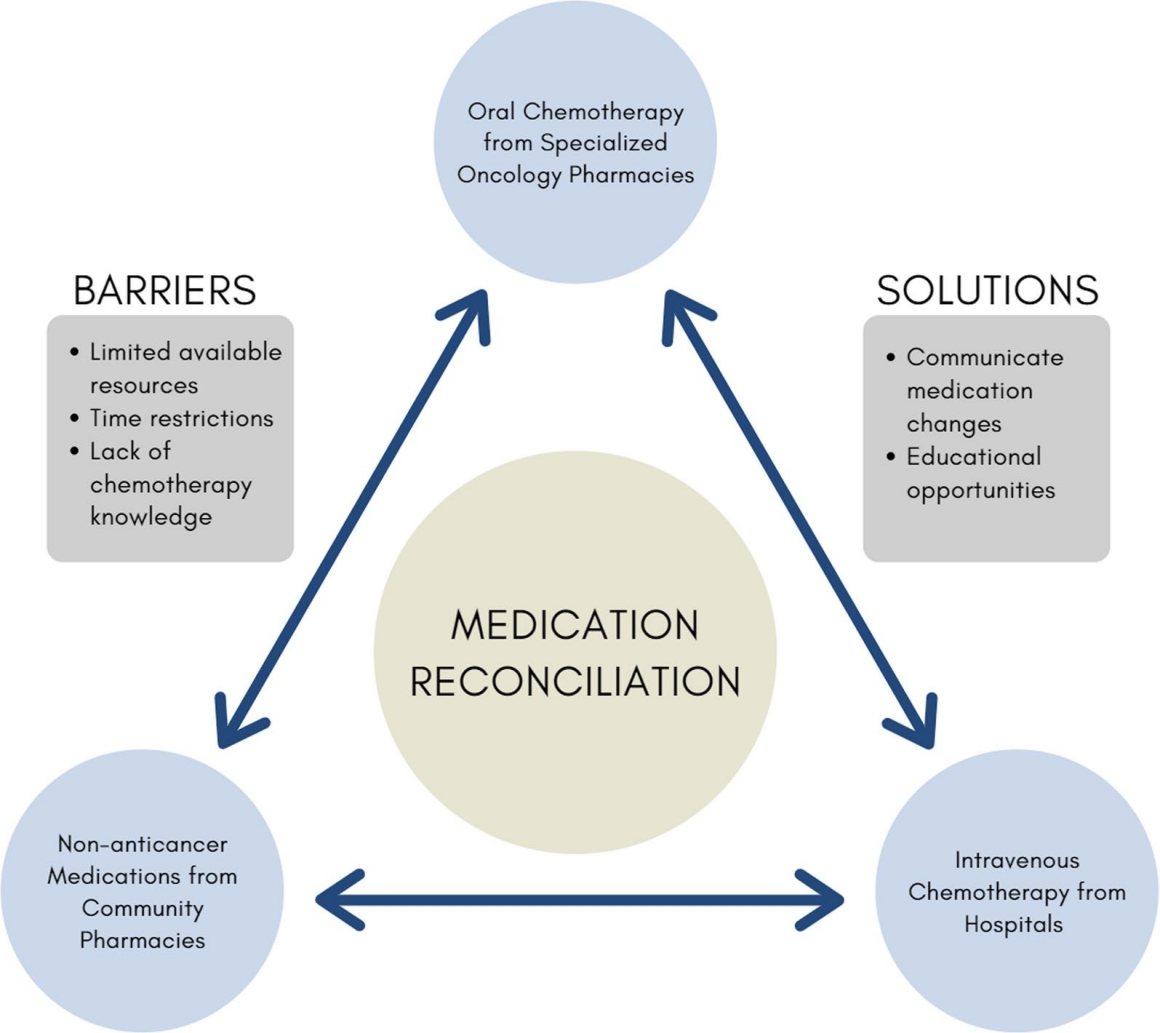
Model of medication reconciliations performed across three potential care settings for oncology patients

Traditionally, chemotherapy is delivered intravenously in inpatient and outpatient hospital settings. Recently, there is an increasing amount of oral chemotherapies being delivered in the community setting. With so many regimens available to treat a variety of cancers, it is not uncommon for oncology patients to receive concurrent intravenous and oral chemotherapies from both hospitals and specialized community pharmacies. For example, a palliative chemotherapy regimen for breast cancer includes oral capecitabine administered twice daily for days 1–14, as well as intravenous trastuzumab on day 1 of each cycle [60]. In addition to receiving anticancer agents, oncology patients may also take medications dispensed routinely from their community pharmacy for their pre-existing conditions and supportive therapies. Patients may find themselves obtaining their medications from multiple locations, which can increase the risk of discrepancies in a patient’s medication record between settings. To ensure continuity of care and patient safety, it is imperative to have an up-to-date medication record and clear communication of decisions between a patient’s primary oncologist, community pharmacist and other health care providers involved. This emphasizes the importance of conducting medication reconciliations, especially in patients that are receiving medications from multiple settings, such as hospitals, specialized oncology pharmacies and community pharmacies, to provide accurate medication management (Fig. 3). Current literature is lacking in this topic, and future studies should investigate advantages from medication reconciliations conducted in patients taking any combination of oral chemotherapy, intravenous chemotherapy, and non-anticancer medications across different pharmacy settings.

Community pharmacists are in an excellent position to support patients taking anticancer medications, since they are often regarded as the most accessible health care providers. However, a study reported that community pharmacists may face barriers to conducting medication reconciliations during transitions from hospital to community care, such as limited resources and time restrictions [61]. Community pharmacists also reported that additional information such as hospital medication discharge lists as well as stop-orders for discontinued medications would be beneficial when conducting medication reconciliations [61]. Similar concepts of ensuring transmission of medication changes across multiple pharmacy settings can be applied to support community pharmacists in conducting medication reconciliations in oncology patients. Another barrier that community pharmacists may face is lack of chemotherapy knowledge. A survey by Abbot et al. found that only 13.6% of community pharmacists felt they had received adequate oncology education at the undergraduate level [62]. Only 24% of pharmacists felt familiar with common doses of oral anticancer agents and only 9% were comfortable counseling patients on these medications [62]. This highlights the need for more educational opportunities to support pharmacists and to ensure confidence and accuracy when reconciling and managing anticancer agents.

## Conclusion

Optimizing medication management in cancer patients may often be overlooked due to the complexity of its nature. Medication reconciliation has been shown to be an essential service that prevents medication errors and ensures medication safety in cancer patients during transitions of care. Medication reconciliations also allows for opportunities to optimize medications through identifying drug interactions, adjusting chemotherapy dosing as well as initiating deprescribing. The clinical impact is evident; however, economic impact is lacking. As more oncology patients receive anticancer medications from multiple settings, it is important to identify discrepancies between them. Future research is warranted to evaluate the benefit of medication reconciliations in oncology patients receiving a combination of oral, intravenous, and non-anticancer medications from multiple sources.

## Data Availability

Data sharing does not apply to this article as no data sets were generated or analyzed during the current study.
